# The Weather

**Published:** 1854-04

**Authors:** 


### THE WEATHER.

The unavoidable delay in the publication of another number of our Journal, vexatious as it is, has this slight advantage, that it enables us to speak of our spring weather, which has been quite remarkable. The "uncertain glories of an April day" have been celebrated in song and have long since passed into a proverb, but we do not remember when this fickle month ever showed so much versatility as it has done in the last fortnight. The wind has been for days from the rawest quarter, cold as December, rendering the flesh as insufficient a protection as if it were thin paper, piercing to the bones, and developing every latent rheumatism and neuralgia in them; and then the soft south wind has prevailed, carrying the thermometer up to summer heat. We have had Fahrenheit's instrument marking eighty degrees one day, and the next day down to the point of freezing. Our latitude exposes us to the two extremes of temperature, the cold of the northern lakes, still bound up in ice, and the heat of tropical seas; and thus we have winter and summer alternately in quick succession according to the direction of the winds. Ships crossing from Liverpool report the appearance of an extraordinary number of icebergs on our eastern coast, encountered during their late voyages, and to these we are doubtless to ascribe something of the raw weather which has characterized the present spring. These vast bodies of ice exert a depressing influence upon the temperature of the air along the shores near which they float; and which is felt far in the interior. Their unusual descent into our southern seas is likely to protract this unseasonable weather, and may yet bring upon us the disastrous frosts which have already visited the Southern States. Severe as the cold has been, we are happy to say that our fruits have thus far escaped, and we may be pardoned for alluding to this fact as important, being, as we are, of the number of those who hold that fruit is highly conducive to human health.

